# Genetic Diversity and Population Genetic Structure Analysis of *Plasmodium knowlesi* Thrombospondin-Related Apical Merozoite Protein (TRAMP) in Clinical Samples

**DOI:** 10.3390/genes13111944

**Published:** 2022-10-25

**Authors:** Md Atique Ahmed, Rehan Haider Zaidi, Gauspasha Yusuf Deshmukh, Ahmed Saif, Mohammed Abdulrahman Alshahrani, Syeda Sabiha Salam, Mohammed Mohieldien Abbas Elfaki, Jin-Hee Han, Saurav Jyoti Patgiri, Fu-Shi Quan

**Affiliations:** 1 ICMR-Regional Medical Research Centre, NER, Dibrugarh, Assam, Bhubaneswar 786010, India; 2 Department of Biotechnology and Microbiology, National College, Tiruchirapalli 620001, India; 3 Department of Clinical Laboratory Sciences, Faculty of Applied Medical Sciences, Najran University, Narjan 55461, Saudi Arabia; 4 Department of Life Sciences, Dibrugarh University, Assam, Dibrugarh 786004, India; 5 Department of Microbiology and Parasitology, Faculty of Medicine, Jazan University, Jizan 45142, Saudi Arabia; 6 Department of Medical Environmental Biology and Tropical Medicine, School of Medicine, Kangwon National University, Chuncheon 24341, Korea; 7 Medical Research Center for Bioreaction to Reactive Oxygen Species and Biomedical Science Institute, School of Medicine, Graduate School, Kyung Hee University, Seoul 02447, Korea; 8 Department of Medical Zoology, School of Medicine, Kyung Hee University, Seoul 02447, Korea

**Keywords:** *Plasmodium knowlesi*, TRAMP, genetic diversity, invasion, population structure, genetic differentiation

## Abstract

The simian malaria parasite *Plasmodium knowlesi* causes a high number of zoonotic infections in Malaysia. The thrombospondin-related apical merozoite protein (TRAMP) is an essential ligand for binding to the erythrocyte cell surface, whereby it facilitates the invasion. This study is the first attempt to determine the genetic diversity, phylogeography, natural selection and population structure from 97 full-length *PkTRAMP* gene sequences originating from Malaysia. We found low levels of nucleotide diversity (π~0.0065) for the full-length gene despite samples originating from geographically separated regions (i.e., Peninsular Malaysia and Malaysian Borneo). The rate of synonymous substitutions was significantly higher than that of non-synonymous substitutions, indicating a purifying selection for the full-length gene within the clinical samples. The population genetic analysis revealed that the parasite population is undergoing a significant population expansion. The analysis of the amino acid sequence alignment of 97 *PkTRAMP* sequences identified 15 haplotypes, of which a major shared haplotype was noted Hap 1 (*n* = 68, Sarawak; *n* = 34, Sabah; *n* = 12, Peninsular Malaysia; *n* = 22). The phylogenetic analysis using DNA sequences identified two clusters that separated due to geographical distance and three mixed clusters with samples from both Peninsular Malaysia and Malaysian Borneo. Population structure analyses indicated two distinct sub-populations (K = 2). Our findings point to the potential for independent parasite evolution, which could make zoonotic malaria control and elimination even more challenging.

## 1. Introduction

Malaria represents one of the most fatal diseases in the developing world, occurring mainly in tropical and sub-tropical regions. Despite considerable advances in our understanding of the disease, malaria continues to be one of the most serious threats to public health, globally contributing to a high morbidity and mortality rate. According to the World Malaria Report 2022, there were an estimated 241 million malaria cases and 627,000 deaths in 2020. Notably, as per the report, there was a significant rise in malaria incidences during the COVID-19 pandemic, thereby affecting various malaria control strategies [1]. Therefore, newer control measures are urgently needed to combat the disease. Among the five *Plasmodium* spp. known to cause human malaria, *P. knowlesi*, a simian malaria parasite, emerged as a major concern in Southeast Asian countries [2,3]; specifically, a high number of cases are being reported from Malaysian Borneo (Sabah and Sarawak) and Peninsular Malaysia [4], thus resulting in malaria researchers continuing to develop effective control measures such as the identification of potential malaria vaccine candidates. Previous *P. knowlesi* whole-genome and genetic studies from clinical samples of Sarawak and Malaysian Borneo revealed *Macaca fascicularis* and *Macaca nemestrina* to be the primary monkey hosts [5,6]. In vitro studies have revealed that the parasite can adapt to both human and macaque RBCs [7]. *P. knowlesi* is known to share microscopic similarities with *P. falciparum, P. malariae* and *P. vivax* [8]. Physiological symptoms of *P. knowlesi* infections include diarrhea, vomiting and multi-organ failure, such as kidney failure, and in extreme cases may even lead to death [9]. *P.* *knowlesi* is an ideal parasite model for malaria research because of its ability to invade primate, i.e., Rhesus macaque, red blood cells (RBCs) [10]. The erythrocytic life-cycle of *P. knowlesi* is very short, being completed in 24 h, a process that otherwise takes 48–72 h in other *P.* species [11]. These rapid developments of parasitemia in the host makes *P. knowlesi* even more life-threatening. The *P.* genome sequencing makes it possible to detect new proteins that have important functions in the parasite life-cycle. Among them are the host-specific cell invasion proteins that trigger the parasite to invade the RBCs and are therefore viewed as important malaria vaccine targets [12]. The invasion of malaria parasites inside the host RBCs is a complex process involving molecular interactions between the parasite ligand and host cell receptor [13]. However, for vaccine development and efficacy, antigenic variation among field isolates is a major challenge. Various genome sequencing studies from clinical samples of *P. knowlesi* exhibited extensive genomic dimorphism [6]. Although various erythrocytic-stage antigens of *P. knowlesi* were studied from clinical samples, e.g., Duffy binding protein (DBP) and several merozoite surface proteins (MSP-1, MSP-1 paralog and MSP-3), none of them were found to be under a strong positive natural selection [5,14]. Therefore, for any merozoite surface protein, it is important to determine the level of polymorphisms, the type of natural selection and its importance, in order to study it as a vaccine candidate. Apicomplexan parasites possess a highly conserved region known as the thrombospondin structural homology repeats (TSR), which plays a major role in host cell invasion. In *Plasmodium* spp., the TSR has been identified and is widely characterized for the thrombospondin-related adhesive protein (TRAP) gene, which is involved in sporozoite gliding motility and mosquito salivary gland invasion. One such important TSR-containing gene is the thrombospondin-related apical merozoite protein (TRAMP), which binds to the host cell surface and increases the binding affinity of the host-ligand association [15]. Located in the merozoites, TRAMP facilitates the entry of parasites into RBCs [16]. *P.* *falciparum* TRAMP is a protein that is present in the organelles present at the apical portion of merozoites within intra-erythrocytic schizonts [17]. Orthologs of this gene have been reported in other *P.* spp., showing the specific conserved function of host cell invasion by the TRAMP gene. Similarly, the orthologs of *Pf*TRAMP have also been observed in *P. vivax*. All this information on the TRAMP gene makes it an ideal malaria vaccine candidate, and yet no prior study has been conducted on the genetic diversity of the *P. knowlesi* *TRAMP* gene. Therefore, the present study aimed to compare the *Pk*TRAMP gene to its orthologs, i.e., *P. vivax*, *P. falciparum* and *P. coatneyi,* and to determine the genetic diversity, natural selection, population structure and phylogeographic analysis of a full-length *PkTRAMP* gene obtained from 97 clinical samples originating from 3 regions of Malaysia, i.e., Sarawak, Sabah from Malaysian Borneo and Peninsular Malaysia.

## 2. Materials and Methods

### 2.1. PkTRAMP Sequence Data

**Figure 1:**
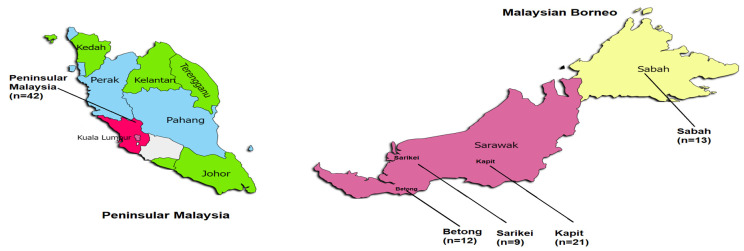
Sample origin from various locations of Malaysia. The *n* represents the number of sequences from each location.

Ninety-seven full length *PkTRAMP* gene sequences originating from Peninsular Malaysia (*n* = 42) and Malaysian Borneo (*n* =55; Betong (*n* = 12), Kapit (*n* =21), Sarikei (*n* = 9) and Sabah (*n* = 13)) were retrieved from public databases (https://www.ncbi.nml.nih.gov (accessed on 23 June 2022)) and (https://www.ebi.ac.uk/ena/browser/home (accessed on 23 June 2022)) from clinical samples (along with the H_strain, PKNH_1437600). A map representing the various geographical regions of the samples used in this study is depicted in Figure 1 and Supplementary Table S1. Signal peptide for the full-length *PkTRAMP* gene was identified using Signal IP 5.0 prediction software [18]. Alignments of sequence data were performed using the CLUSTAL-W program in MegAlign Lasergene v 7.0 (DNASTAR, Madison, WI, USA) software. To determine the relationship among the *PkTRAMP* sequences (clinical samples from Malaysian Borneo and Peninsular Malaysia), a phylogenetic analysis was performed using nucleotide sequences by using the Maximum Likelihood (ML) method with 1000 bootstraps in MEGA 5.0 software [19]. To determine the evolutionary relationships, TRAMP ortholog sequences in *P. falciparum* (PF3D7_1218000), *P. vivax* (PVX_123575), *P. coatneyi* (PCOAH_00055120) and *P. knowlesi* H-strain (PKNH_1437600) were also included.

### 2.2. Sequence Diversity and Natural Selection

DnaSP v5.10 software was used to study the sequence diversity (π) [20]. The software was also used to evaluate the number of polymorphic sites, synonymous substitutions (silent mutations) and nonsynonymous substitutions (replacement change), singletons (a variant of nucleotide that appears once in sequence), the number of haplotypes (H), parsimony informative sites (sites with a minimum of two nucleotides that appear at least twice) and haplotype diversity for the full-length *Pf*TRAMP gene [20] along with the reference strain H_strain, PKNH_1437600. Nucleotide diversity was graphically represented using a window length of 80 bp and a step size of 15 bp in DnaSP software. Polymorphism and phylogenetic analyses were performed in MEGA 5.0 software [19].

Furthermore, to determine natural selection, Nei and Gojobori’s method was used to compute the rate of nonsynonymous substitutions per nonsynonymous site (dN) and the rate of synonymous substitutions per synonymous site (dS). In addition to this, to calculate the natural selection, other analyses such as Tajima’s D, Fu & Li’s D* and F* neutrality tests were also conducted using the DnaSP v5.10 software. Under neutrality, Tajima’s D value is expected to be zero. A significant and positive value of Tajima’s D represents positive/balancing selection, while a negative value indicates negative selection or population expansion. Furthermore, DnaSP software was also used to represent the graphical illustration of Tajima’s D values. Significant positive values of Fu & Li’s D* and F* indicate population contraction, while negative values indicate population expansion and an excess of singletons.

### 2.3. Population Structure and Genetic Differentiation Analysis

To estimate the population structure of *P. kTRAMP*, STRUCTURE v 2.3.4 software was used [21]. An admixture model was selected within the software to determine the most likely number of populations (K). For each population, K was set from 1 to 10 and a single run was conducted with 15 iterations. A Markov Chain Monte Carlo generation of 500,000 was used for each run following a burn-in period of 50,000 steps. The STRUCTURE Harvester [22] online server was used to estimate the most likely K-value by computing ΔK values that were based on the rate of change in log probability (LnP(D)) between consecutive K-values.

ARLEQUIN version 3.5.1.3 software (University of Berne, Bern, Switzerland) [23] was further used to compute the pair-wise differences (F_ST_) between *P. knowlesi* parasite populations originating from Peninsular Malaysia and Malaysian Borneo. F_ST_ values can be interpreted as no genetic differentiation when the value is 0, lower when <0–0.05, moderate when 0.05–0.15, and high differentiation when F_ST_ is 0.15–0.25.

## 3. Results

### 3.1. PkTRAMP Sequence Identity within Ortholog members and Diversity within P. knowlesi Population

**Figure 2:**
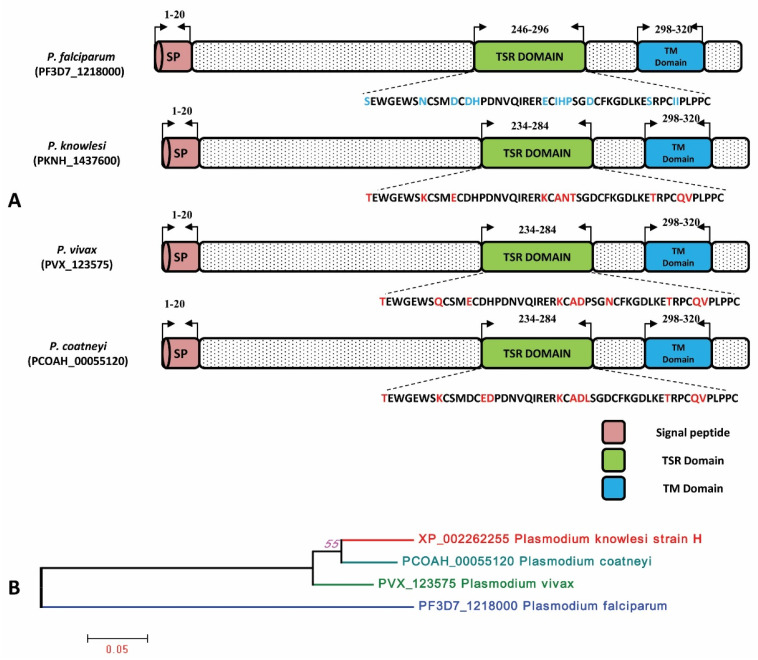
(**A**) Schematic structure of TRAMP gene in *P. falciparum, P. knowlesi*, *P. vivax* and *P. coatneyi*. The Signal peptide, TSR and TM domain have been marked by the arrows with their sequence positions. The amino acid polymorphisms in the TSR domain within three *Plasmodium* sp. are highlighted in red in comparison to *P. falciparum*. SP: Signal peptide, TSR: Thrombospondin type-1 repeat super family, TM: Transmembrane. (**B**) Phylogenetic tree of *TRAMP* gene constructed using MEGA 5.0 by ML method shows inter-species relation between *P. knowlesi*, *P. coatneyi*, *P. vivax* and *P. falciparum*.

The signal peptide of the PkTRAMP protein was identified between amino acid positions 1 and 20, as shown in Supplementary Figure S1. The analysis of amino acid sequences revealed that the *P. knowlesi* TRAMP (reference H strain) protein has 90.29%, 88.24% and 61.95% sequence identities with its orthologs in *P. coatneyi, P. vivax* and *P. falciparum,* respectively. The schematic representations of the TRAMP gene structure and inter-species amino acid polymorphism were observed in the thrombospondin structural homology repeat (TSR) domain and transmembrane domain (TM) and are shown in Figure 2A. The inter-species phylogenetic relationships between TRAMP orthologs are shown in Figure 2B.

### 3.2. Amino Acid Haplotype

**Figure 3:**
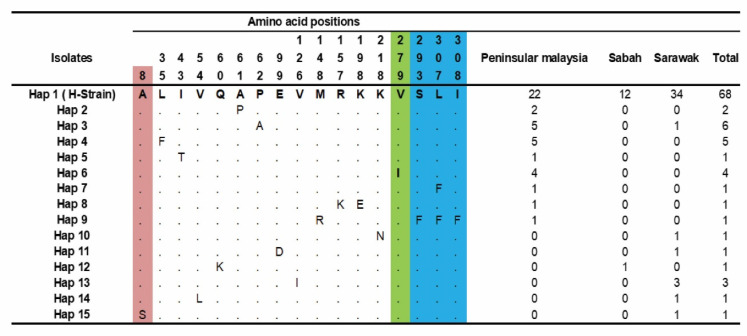
Amino acid haplotypes observed within PkTRAMP from Malaysian samples. Amino acids identical to reference strain H (PKNH_1436700) are indicated in the top row Hap 1. The maroon color indicates the SP domain, green color indicates the TSR domain and blue color indicates the TM domain. Total number of haplotypes indicated in right panel. Dots represent identical amino acid, and the numbers in vertical represent the position of the amino acids with respect to the H-strain.

The amino acid polymorphism analysis from 97 clinical samples detected 15 haplotypes, as shown in Figure 3. Haplotype-1 (H-strain) was found to be the major haplotype (*n* = 68), with the highest frequency in Sarawak (*n* = 34), followed by Peninsular Malaysia (*n* = 22) and Sabah (*n* = 12). Furthermore, minor haplotypes were observed in Hap-3 (*n* = 6), Hap-4 (*n* = 5) and Hap-6(*n* = 4), where the majority of the sequences represented came from Peninsular Malaysia.

### 3.3. Nucleotide Diversity and Polymorphisms

The sequence analysis of the full-length *PkTRAMP* gene (*n* = 97) identified 53 polymorphic sites, of which 36 were synonymous and 17 were nonsynonymous substitutions sites. Among these, 23 sites were from two variants, 69 haplotypes with a haplotype diversity 0.99 ± 0.003 (Table 1). The overall nucleotide diversity of 97 full-length *PkTRAMP* was found to be π = 0.00652 ± 0.00028 (Table 1). The region-wise analysis of 97 clinical samples, i.e., Peninsula Malaysia (*n* = 42), Sarawak (*n* = 42) and Sabah (*n* = 13), is shown in Table 1. The nucleotide diversity of Peninsular Malaysia (π = 0.00662 ± 0.00035) is found to be greater than that of Sabah (π = 0.00615 ± 0.00051) and Sarawak (π = 0.00457 ± 0.00029) (Table 1). The haplotype diversity values of Peninsular Malaysia, Sabah and Sarawak are found to be in the same range (Table 1). In these three locations, the rate of synonymous substitutions is found to be higher. The higher number of singleton sites within TRAMP gene samples originating from Peninsular Malaysia and Sarawak has led to a higher amount of haplotype diversity and number of haplotypes, as shown in Table 1.

### 3.4. Natural Selection in PkTRAMP

**Figure 4:**
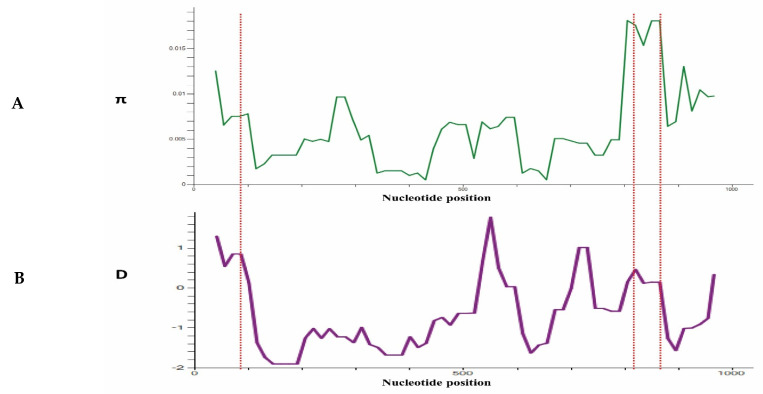
(**A**) Graphical representation of nucleotide diversity (π) within 97 sequences (1001 bp) of *PkTRAMP* gene from Malaysian clinical isolates. The probable epitopes are marked with a star symbol. (**B**) Graphical representation of Tajiam’s D value across the *PkTRAMP* gene. Red dotted lines are used to indicate the peaks in the (π) graph and D graph.

To determine whether natural selection contributes to polymorphism in the *PkTRAMP* full-length gene, multiple tests were conducted at the intra-species level. The full-length gene analysis of 97 sequences showed negative values for dS-dN, Tajimas’s D and Fu & Li D* and F*, indicating a purifying selection and population expansion (Table 1). The values obtained for Fu & Li D* are significant for the overall samples and Sarawak samples (Table 1). A graphical representation of the nucleotide diversity and Tajima D values, indicating regions of high diversity (showing peaks) within the gene, is shown in Figure 4A,B.

### 3.5. Phylogenetic Analysis

**Figure 5:**
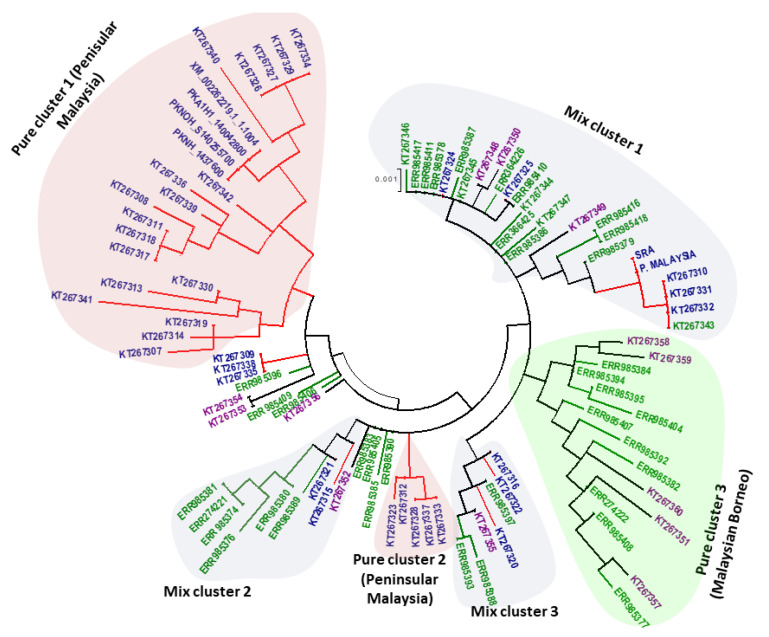
Phylogenetic tree comprising 97 nucleotide sequences of the *PkTRAMP* gene generated by MEGA 5.0 using the Maximum Likelihood method. The constructed tree shows clustering of the *PkTRAMP* gene from different regions and is labeled as pure clusters 1 & 2, which contain samples from Peninsular Malaysia (light pink), pure cluster 3, which contains samples from Malaysian Borneo (light green), and mixed clusters 1, 2 and 3, which contain samples from both Malaysian Borneo and Peninsular Malaysia (light grey).

The phylogenetic analysis of 97 full-length *PkTRAMP* nucleotide sequences using the ML method showed an intermixed clustering of samples along with some pure clusters that constituted samples originating from a single region. The clusters were named as pure cluster and mixed cluster. Three pure clusters were identified, of which two included samples from Peninsular Malaysia (pure clusters 1 & 2) and one from Malaysian Borneo (pure cluster 3), as were three mixed clusters, which constituted samples from different regions of Malaysia (Figure 5). Overall, the phylogenetic tree indicated the presence of two types of parasite populations.

### 3.6. Genetic Differentiation and Population Structure

**Figure 6:**
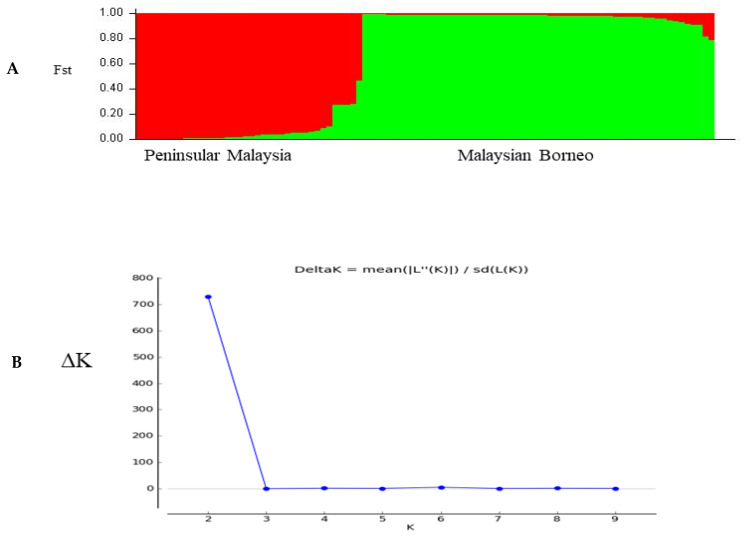
(**A**) Most likely number of parasite sub-populations (K = 2) based on PkTRAMP gene within 97 samples from different regions of Malaysia. (**B**) The number of sub-populations was estimated by the delta K value (ΔK), and two distinct sub-populations were observed (K = 2, ΔK = 730). The line graph of population structure is shown using membership coefficients (Q values).

The population structure of the three distinct geographical regions was estimated using pair-wise genetic differentiation (F_ST_) values using ARLEQUIN software version 3.5.1.3. A moderate level of genetic differentiation was observed between samples from Sarawak and Sabah (F_ST_ = 0.01146; *p* < 0.05), followed by Peninsular Malaysia and Sabah (F_ST_ = 0.00045; *p* < 0.05), as shown in Table 2. Significant differentiation values were observed between the considered regions. Bayesian inference produced the two most significant genetic populations based on their significant K value (K = 2), indicated in red and green colors (Figure 6A) and the corresponding ∆K =730 values are shown in (Figure 6B).

## 4. Discussion

Located at the merozoite surface, blood-stage antigens play a significant role in the parasite invasion of the host RBC membrane. During merozoite egress from the liver cells, these antigens, being directly exposed to host defensive immunity, represent an important vaccine candidate. In the context of *P. knowlesi*, no study has been conducted to genetically characterize the *PkTRAMP* gene. This study is perhaps a preliminary attempt, involving the comparative analysis of the *P. knowlesi* TRAMP gene with its orthologous sequences, which are *P. falciparum*, *P. vivax* and *P. coatneyi,* in order to understand the antigenic variability, natural selection and population structure in clinical samples from Malaysia. *P.* *knowlesi* protein PkTRAMP contains Thrombospondin structural repeats (TSR) and the transmembrane domain. At various levels of the parasite’s life cycle, the TSR domain plays an important role in the molecular interaction between the parasite and diverse host tissues, guiding motility and host cell recognition [12]. The characterization of the TSR domain showed that its expression in *P. knowlesi* merozoites implicated its importance in erythrocytic cell invasion [16]. In the case of *P. falciparum*, the TRAMP gene is proteolytically cleaved and then released during the host cell invasion in the erythrocytic cell [17]. The main aim of this study was to genetically characterize the *PkTRAMP* gene from clinical samples from Sarawak, Sabah and Peninsular Malaysia, where a high number of cases of *P. knowlesi* is being reported. The low level of polymorphism in the TSR domain revealed from the inter-species analysis of the TRAMP gene indicates that it might be an excellent candidate; however, immunological and functional studies need to be conducted. The amino acid alignment analysis of *Pf*TRAMP and *Pv*TRAMP revealed that both species share approximately a 88.2% identity, indicating the potential use of this antigen for vaccine development [12,15]. Moreover, the amino acid sequence analysis of *P. knowlesi* reference H-strain was found to have 90.29%, 88.24% and 61.95% sequence identities with its orthologs *P. coatneyi*, *P. vivax* and *P. falciparum,* respectively. A comparison of amino acid haplotypes of PkTRAMP revealed that one major haplotype (Haplotype-1) had the highest frequency with samples from all regions of Malaysia.

The test for the natural selection analysis (dS-dN) indicated that the PkTRAMP gene was under negative selection. Furthermore, the negative values of other population genetic analysis, e.g., Taj’s D and Li & Fu’s D* and F*, were also indicative of PkTRAMP being under purifying selection. The significant values for Li & Fu’s D* and F* were also indicative of population expansion, owing to a high number of singleton sites. The graphical analysis of the nucleotide diversity and Taj’s D values showed the highest peaks towards the end of the 3′-region (nucleotide positions: 800–900), which had the highest Taj’D value, implicating a higher degree of polymorphism compared to the rest of the gene. Several similar studies with a strong negative selection pressure on *P. knowlesi* merozoite surface antigens have been reported [5,24,25,26,27,28].

The phylogenetic analysis’s findings revealed that among the 97 PkTRAMP, some clusters formed as a result of geographic clustering, but host-specific clustering resulting from adaptation to the natural hosts Macaca fascicularis and Macaca nemestrina, as identified by other previous studies [24,25,27], was not noted. Three pure clusters (geographical clustering) were identified, i.e., clusters 1, 2 and 3, two of which originated from Peninsular Malaysia and one from Malaysian Borneo. These clusters observed in the phylogenetic analysis may be due to the adaption and population expansion of the parasite to the primary hosts, i.e., the macaque species. This was represented by the genetic diversity in spite of samples being collected from the same location. However, one should note that a robust population genetic structure analysis using STRUCTURE revealed that only two major sub-populations (K = 2) exist in Malaysia, similar to previous studies [29].

## 5. Conclusions

This is the first study to determine the level of polymorphism, natural selection and population structure of the *PkTRAMP* gene from clinical samples from Malaysia. The low level of genetic diversity observed within the *PkTRAMP* sequences could be helpful to vaccine-oriented studies; however, further immunological and functional studies are necessary. The multiple pure and mixed clusters identified in this study were indicative of sub-populations coexisting in the regions, with a population expansion being evident. Future studies should investigate the genetic diversity of this gene among *P. knowlesi* samples from all Southeast Asian countries.

## Figures and Tables

**Table 1:** Estimates of nucleotide diversity, haplotype diversity and neutrality indices.

Diversity ± SD								Codon-Based z- Test dS-dN	Fu & Li’s D*	Fu & Li’s F*	Taj D
Location	No. of Samples	SNPs	Syn.	NonSyn.	No. of Haplotypes	Haplotype	Nucleotide				
P. Malaysia	42	30	19	11	27	0.971 ± 0.012	0.00662 ± 0.00035	3.91	−0.21	−0.24	−0.17
Sabah	13	20	19	1	12	0.987 ± 0.035	0.00615 ± 0.00051	4.03	−0.45	−0.43	−0.19
Sarawak	42	29	23	6	33	0.984 ± 0.010	0.00457 ± 0.00029	3.56	−2.62 ^#^	−2.48	−1.09
Overall	97	53	36	17	69	0.99 ± 0.003	0.00652 ± 0.00028	4.26	−2.86 ^#^	−2.61 ^#^	−1.17

^#^ indicates significant values, i.e., *p* < 0.05.

**Table 2:** Population differentiation values (FST) for *PkTRAMP* from Peninsular Malaysia and Malaysian Borneo.

Locations	Fst Values		
	Peninsular Malaysia	Sabah	Sarawak
Peninsular Malaysia	-	-	-
Sabah	0.00045 ± 0.0002 *	-	-
Sarawak	0.00038 ± 0.0003 *	0.01146 ± 0.002 *	-

* *p* < 0.05.

## Data Availability

Publicly available datasets were analyzed in this study. The repository is ENA (European nucleotide archive: https://www.ebi.ac.uk/ena/browser/home (accessed on 23 June 2022)) and https://www.ncbi.nml.nih.gov (accessed on 23 June 2022). Accession numbers can be found in the Supplementary Material.

## Supplementary Materials

The following supporting information can be downloaded at: https://www.mdpi.com/article/10.3390/genes13111944/s1, Table S1: Study samples (accession numbers) and location/orgin; Figure S1: Study samples (accession numbers) and location/orgin.
