# Supplementary material for: Genetic Diversity and Population Genetic Structure Analysis of Plasmodium knowlesi Thrombospondin-Related Apical Merozoite Protein (TRAMP) in Clinical Samples
Source: Genes (Basel). 2022 Oct 25;13(11):1944. doi: 10.3390/genes13111944 (PMC9689803; doi:10.3390/genes13111944)

Supplementary Table S1. Study samples(accession numbers) and location/orgin.

S.no	Sample I'd	Location
1	XM_002262219	P. Malaysia
2	SRA (Philippine strain)	P. Malaysia
3	P.MALAYSIA (MR4)	P. Malaysia
4	PKA1H1_140042800	P. Malaysia
5	PKNH_1437600	P. Malaysia
6	PKNOH_S140255700	P. Malaysia
7	KT267307	P. Malaysia
8	KT267308	P. Malaysia
9	KT267309	P. Malaysia
10	KT267310	P. Malaysia
11	KT267311	P. Malaysia
12	KT267312	P. Malaysia
13	KT267313	P. Malaysia
14	KT267314	P. Malaysia
15	KT267315	P. Malaysia
16	KT267316	P. Malaysia
17	KT267317	P. Malaysia
18	KT267318	P. Malaysia
19	KT267319	P. Malaysia
20	KT267320	P. Malaysia
21	KT267321	P. Malaysia
22	KT267322	P. Malaysia
23	KT267323	P. Malaysia
24	KT267324	P. Malaysia
25	KT267325	P. Malaysia
26	KT267326	P. Malaysia
27	KT267327	P. Malaysia
28	KT267328	P. Malaysia
29	KT267329	P. Malaysia
30	KT267330	P. Malaysia
31	KT267331	P. Malaysia
32	KT267332	P. Malaysia
33	KT267333	P. Malaysia
34	KT267334	P. Malaysia
35	KT267335	P. Malaysia
36	KT267336	P. Malaysia
37	KT267337	P. Malaysia
38	KT267338	P. Malaysia
39	KT267339	P. Malaysia

40	KT267340	P. Malaysia
41	KT267341	P. Malaysia
42	KT267342	P. Malaysia
43	KT267343	Sarikei
44	KT267344	Sarikei
45	KT267345	Sarikei
46	KT267346	Sarikei
47	KT267347	Sarikei
48	KT267348	Sabah
49	KT267349	Sabah
50	KT267350	Sabah
51	KT267351	Sabah
52	KT267352	Sabah
53	KT267353	Sabah
54	KT267354	Sabah
55	KT267355	Sabah
56	KT267356	Sabah
57	KT267357	Sabah
58	KT267358	Sabah
59	KT267359	Sabah
60	KT267360	Sabah
61	ERR274221	Sarikei
62	ERR274222	Sarikei
63	ERR366425	Sarikei
64	ERR364226	Sarikei
65	ERR985374	Betong
66	ERR985376	Betong
67	ERR985377	Betong
68	ERR985378	Betong
69	ERR985379	Betong
70	ERR985380	Betong
71	ERR985381	Betong
72	ERR985382	Betong
73	ERR985383	Betong
74	ERR985384	Betong
75	ERR985410	Betong
76	ERR985411	Betong
77	ERR985385	Kapit
78	ERR985386	Kapit
79	ERR985387	Kapit
80	ERR985388	Kapit
81	ERR985389	Kapit
82	ERR985390	Kapit

83	ERR985392	Kapit
84	ERR985393	Kapit
85	ERR985394	Kapit
86	ERR985395	Kapit
87	ERR985396	Kapit
88	ERR985397	Kapit
89	ERR985404	Kapit
90	ERR985405	Kapit
91	ERR985406	Kapit
92	ERR985407	Kapit
93	ERR985408	Kapit
94	ERR985409	Kapit
95	ERR985416	Kapit
96	ERR985417	Kapit
97	ERR985418	Kapit

Figure S1: Study samples (accession numbers) and location/orgin.

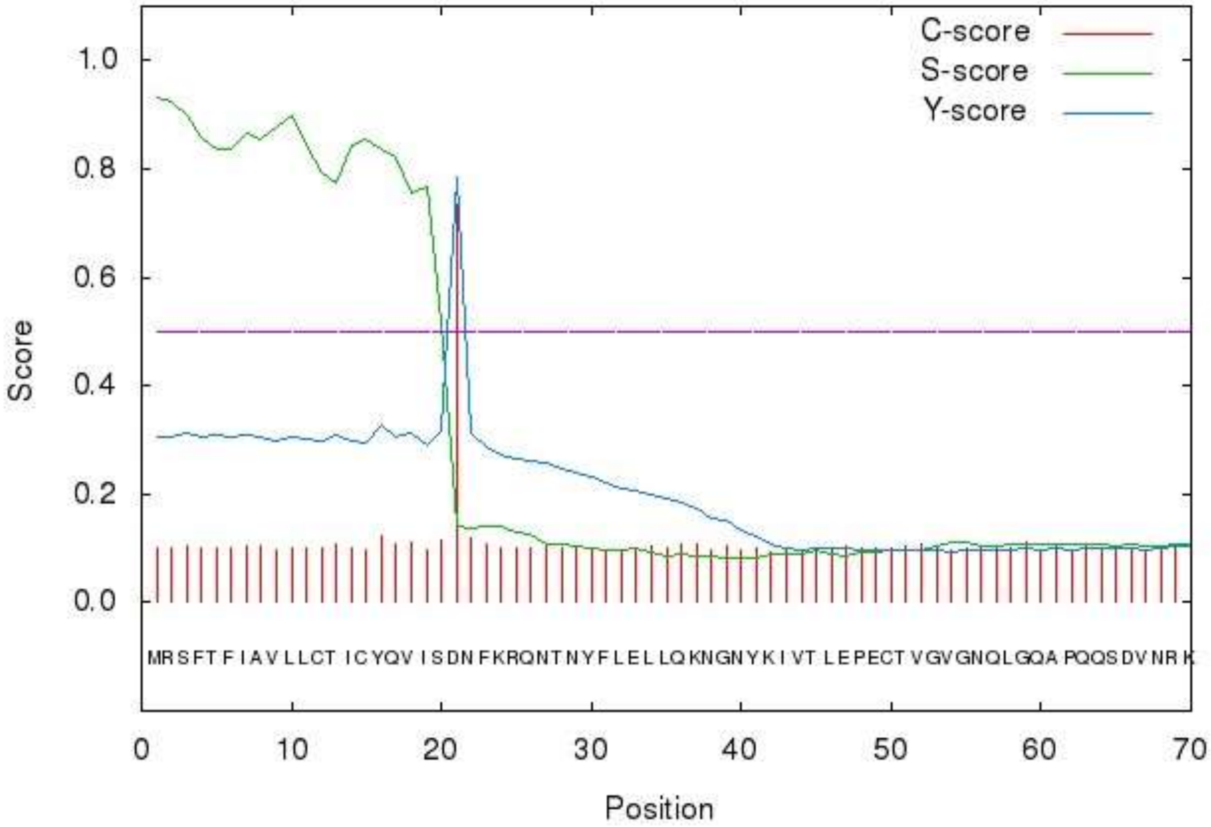

Supplement: Supplementary file 1 [file genes-13-01944-s001.zip › genes-1960915-supplementary.pdf]
